# Discrepancies between genetic and ecological divergence patterns suggest a complex biogeographic history in a Neotropical genus

**DOI:** 10.1002/ece3.6227

**Published:** 2020-04-01

**Authors:** Giorgio Binelli, William Montaigne, Daniel Sabatier, Caroline Scotti‐Saintagne, Ivan Scotti

**Affiliations:** ^1^ DBSV Università dell'Insubria Varese Italy; ^2^ UMR EcoFoG Université des Antilles et de la Guyane Kourou French Guiana; ^3^ AMAP IRD CIRAD CNRS INRAE Université de Montpellier Montpellier France; ^4^ INRAE URFM Avignon France

**Keywords:** allopatric divergence, Amazon, Guiana Shield, interspecific gene flow, Myristicaceae, secondary contact, Virola

## Abstract

Phylogenetic patterns and the underlying speciation processes can be deduced from morphological, functional, and ecological patterns of species similarity and divergence. In some cases, though, species retain multiple similarities and remain almost indistinguishable; in other cases, evolutionary convergence can make such patterns misleading; very often in such cases, the “true” picture only emerges from carefully built molecular phylogenies, which may come with major surprises. In addition, closely related species may experience gene flow after divergence, thus potentially blurring species delimitation. By means of advanced inferential methods, we studied molecular divergence between species of the *Virola* genus (Myristicaceae): widespread *Virola michelii* and recently described, endemic *V. kwatae*, using widespread *V. surinamensis* as a more distantly related outgroup with different ecology and morphology—although with overlapping range. Contrary to expectations, we found that the latter, and not *V. michelii*, was sister to *V. kwatae*. Therefore, *V. kwatae* probably diverged from *V. surinamensis* through a recent morphological and ecological shift, which brought it close to distantly related *V. michelii*. Through the modeling of the divergence process, we inferred that gene flow between *V. surinamensis* and *V*. *kwatae* stopped soon after their divergence and resumed later, in a classical secondary contact event which did not erase their ecological and morphological differences. While we cannot exclude that initial divergence occurred in allopatry, current species distribution and the absence of geographical barriers make complete isolation during speciation unlikely. We tentatively conclude that (a) it is possible that divergence occurred in allopatry/parapatry and (b) secondary contact did not suppress divergence.

## Graphical abstract

We studied molecular divergence between species of the Neotropical *Virola* genus: widespread *Virola michelii* and recently described, endemic *V. kwatae*, using widespread *V. surinamensis* as a more distantly related outgroup with different ecology and morphology—although with overlapping range. We found that the latter, and not *V. michelii*, is sister to *V. kwatae*. This partial reassessment of the phylogeny in the *Virola* genus led us to investigate and to assess, by using coalescent theory and Bayesian modelling, the presence of gene flow after speciation in the pair *V. kwatae*/*V. surinamensis*, indicating the existence of phylogenetic radiation without strict reproductive isolation.

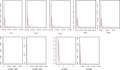

## 1 INTRODUCTION

The term “Ecological speciation” refers to a speciation process in which ecological divergence precedes, and causes, reproductive isolation, implying that at least the early stages of ecological divergence occur in presence of gene flow (Nosil, 2012; Rundle & Nosil, 2005). In plants, this has been observed in model species such as poplar (Christe et al., 2017 and ref. therein), sunflower (Renaut, Owens, & Rieseberg, 2014), and spruce (de Lafontaine, Prunier, Gérardi, & Bousquet, 2015). That said, even though natural interspecific gene flow commonly occurs in flowering plants (Mallet, 2005), including tropical trees (Leigh et al., 2004), species integrity can persist unaffected (Petit & Hampe, 2006). In other instances, gene flow can hamper divergence and lead to cases of incomplete speciation (Mallet, 2008). Gene flow among otherwise well‐defined species occupies a special place in evolutionary biology, and genera including closely related species sharing genetic variants through gene flow—as opposed to sharing alleles inherited from their common ancestor—deserve the special name of “species complexes”. Cases involving ecologically divergent species (i.e., species with nonoverlapping ecological niches) are particularly interesting because they show that ecological radiation may occur, or be maintained, in spite of gene flow (Givnish, 2010; Humphries & Winker, 2011; Nosil, Egan, & Funk, 2008; Nosil, Funk, & Ortiz‐Barrientos, 2009; Petit, Bodénès, Ducousso, Roussel, & Kremer, 2004; Zheng & Ge, 2010). There are currently only few described cases of (ecological) divergence with gene flow, and the frequency of such events is still a matter of debate (Nosil, 2008).

Molecular methods are useful to recount the evolutionary history of species and more specifically to detect interspecific gene flow. Highly polymorphic genetic markers, often in a combination of neutral nuclear and chloroplast markers (e.g., microsatellites) and powerful statistical methods (e.g., Bayesian clustering) facilitate this task (Field, Ayre, Whelan, & Young, 2010). In theory, because they have different evolutionary dynamics (Powell, 1983), chloroplast and nuclear DNA should provide complementary information. Such data can help to describe gene flow and speciation processes (Lexer & Widmer, 2008) and to detect hybrid individuals (Rieseberg & Soltis, 1991). Gene flow is difficult to distinguish from shared ancestral variation as both scenarios produce similar patterns of allele sharing (Muir & Schlötterer, 2005; Won & Hey, 2005). The coalescent theory (Kingman, 1982) offers a strategy to disentangle the effects of the different forces that led to the currently observed patterns of molecular diversity (Bowie, Fjeldså, Hackett, Bates, & Crowe, 2006; Rosenberg & Nordborg, 2002). Divergence with gene flow is suitably inferred by Approximate Bayesian Computation (ABC; Bertorelle, Benazzo, & Mona, 2010; Csilléry, Blum, Gaggiotti, & François, 2010). From the seminal paper on maize by Ross‐Ibarra, Bernatchez, & Gagnaire (2009), only a few studies have used modeling to assess ecological speciation scenarios; after Strasburg and Rieseberg (2013), who advocate for further development of analytical approaches for distinguishing primary from secondary contact, several works on this topic have been published, focused on model species such as the European oak species complex (Leroy et al., 2017) and fish species (Rougeux, Bernatchez, & Gagnaire, 2017).

We have studied the intensity of gene flow during and after speciation through a combination of coalescent modeling and ABC inference in the Neotropical *Virola* genus, which shows a large ecological and species diversity (Wilson, 2004). The resolution of *Virola* phylogeny is not so well‐defined (Steeves, 2011), suggesting that some groups of species are related closely enough that they may be connected by interspecific gene flow. Given that some clades with poor resolution include species with contrasting ecological preferences, this genus is a good model to assess the amount of interspecific gene flow occurring between closely related, ecologically divergent species. An opportunity to do this is provided by the Guiana shield, the northernmost stable part (or *craton*) forming the South American tectonic plate and by a sympatric trio of species of the *Virola* genus thriving there: *terra firme*‐dwelling *V. michelii* Heckel and *V. kwatae* Sabatier, morphologically and ecologically very similar to each other (*terra firme* being the rainforest that is not inundated by flooded rivers), and the much more distinct *V. surinamensis* Warburg, which occupies seasonally flooded areas in combination with the former two species (Baraloto, Morneau, Bonal, Blanc, & Ferry, 2007; Macía, 2011; Sabatier, 1997). Until recently, herbarium vouchers (CAY, P, NY, INPA) of *V. kwatae* were identified as *V. michelii*; nowadays, based on morphological traits, *V. kwatae* is recognized as an independent species (Sabatier, 1997). Because of the very close morphological and ecological resemblance of *V. kwatae* to *V. michelii*, we set out to assess whether the two species evolved with sustained gene flow, and used the third co‐occurring species of the same *Virola* sub‐clade, *V. surinamensis*, as an outgroup. The preliminary results we obtained (see Section 3) forced us to review the relationships among the tree species: it turned out that, in spite of sizeable morphological differences and distinct ecology, *V. surinamensis*, and not *V. michelii*, is a sister species to *V. kwatae*. This implies that the divergence between the two sister species involved at least one ecological shift. Did the two sister species diverge, both morphologically and ecologically, while undergoing gene flow? We considered three scenarios: (a) speciation‐with‐gene‐flow (Feder, Egan, & Nosil, 2012), implying that gene flow was concomitant with speciation; (b) secondary contact, according to which gene flow would have resumed after completion of divergence, which would be maintained in spite of secondary gene flow (Barton & Hewitt, 1985); (c) divergence without gene flow.

## 2 MATERIAL AND METHODS

### 2.1 Species ecology and phylogenetic relationships

The *Virola* genus belongs to Myristicaceae (the nutmeg family), a widely distributed pantropical plant family, member of Magnoliales, and one of the oldest families of flowering plants (Cronquist, 1981; Doyle, Manchester, & Sauquet, 2008; Doyle, Sauquet, Scharaschkin, & Le Thomas, 2004). The Neotropical genus *Virola* is one of ten most common Amazonian tree genera in order of abundance (ter Steege et al., 2013), comprising between 45 (Wilson, 2004) and 60 (Steeves, 2011) species and having its center of diversity in Western Amazonia (Holbrook, Loiselle, & Clark, 2006; Queenborough, Burslem, Garwood, & Valencia, 2007). The genus' phylogenetic resolution is incomplete (Steeves, 2011). *Virola* species are relatively common dioecious canopy trees in the Guiana shield and throughout the Amazon (ter Steege et al., 2013). According to data from 76 1‐ha plots inventoried in French Guiana (Guyadiv database, http://vmcebagn‐dev.ird.fr, D. Sabatier & J‐F. Molino, unpublished), average population densities of *V. surinamensis*, *V. kwatae* and *V. michelii* are about 0.1, 0.5, and 3 adult trees/ha, with up to 7, 5, and 12 trees in a single plot, respectively. These three sympatric species have overlapping phenology patterns; they mostly flower during the dry season (July to November) and fruits ripen during the rainy season (November to June; Sabatier, 1997; ter Steege & Persaud, 1991). They are insect‐pollinated and have vertebrate‐dispersed seeds. Seeds have a limited dormancy, and seedlings form large cohorts growing quickly in forest gaps; surviving saplings become upper‐canopy or emerging trees (Rodrigues, 1980). *V. michelii* and *V. kwatae* are *terra firme* forest specialists, while *V. surinamensis* is found in seasonally flooded bottomland forests (Baraloto et al., 2007; Sabatier, 1997). Until recently, specimens of *V. kwatae* were identified as *V. michelii* in the herbaria and floras. Although these two species occupy the same *terra firme* forest environment, some morphological characters (such as leaf shape, abaxial lamina indumentum, fruit size and indumenta and tree and buttress stature) eventually permitted to distinguish them, leading to the description of *V. kwatae* as a novel species (Sabatier, 1997). Moreover, *V. kwatae* fruits and seeds are always significantly larger than those of its two congeners (size coefficients of seed length: 1.3 and 1.5 compared to *V. michelii* and *V. surinamensis*, respectively); a feature probably linked to its high dispersal rate by spider monkeys (Sabatier, 1997).

### 2.2 Sampling and DNA extraction

**FIGURE 1:**
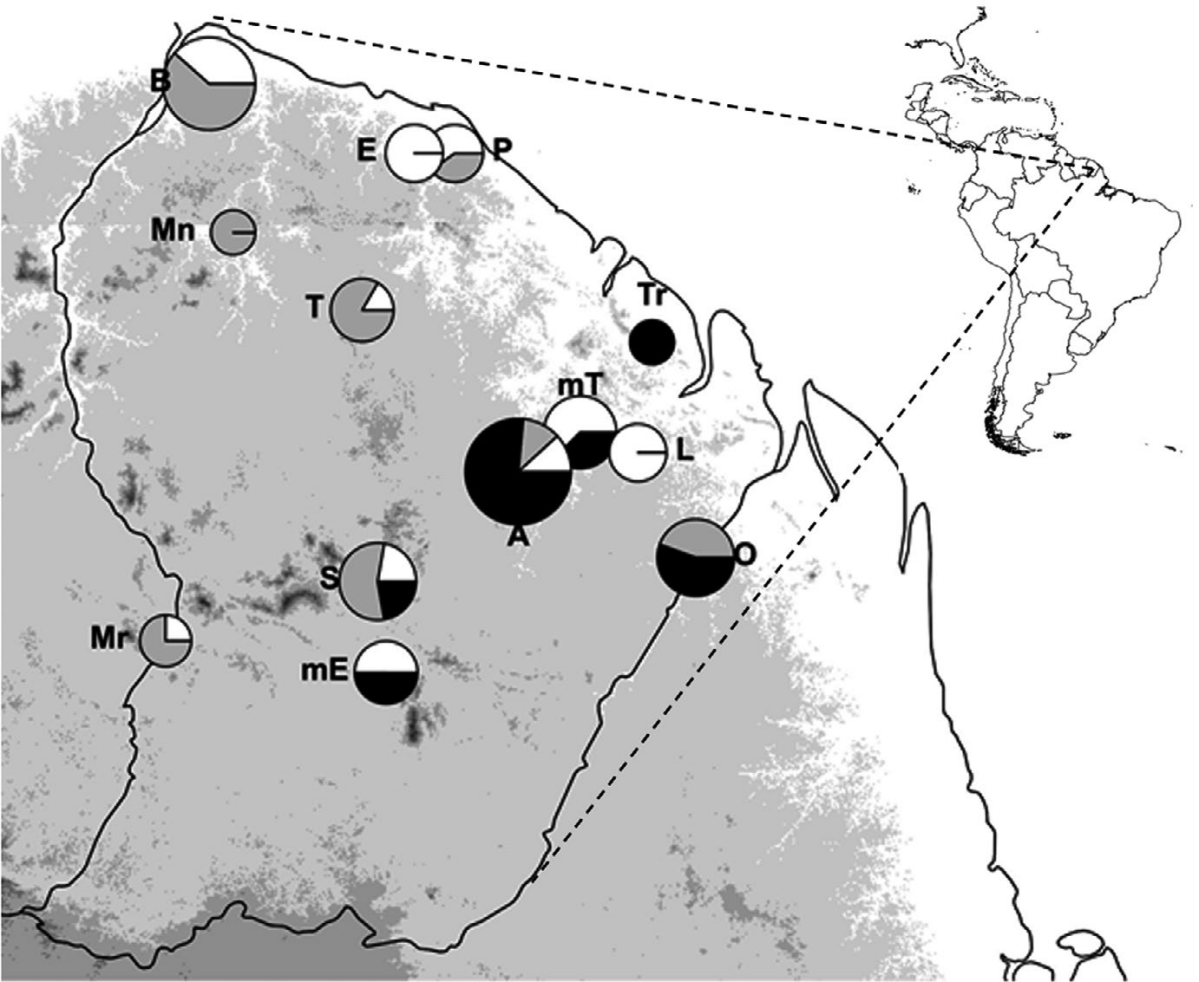
Map of French Guiana, showing the sampling sites of the trees used in this study. The position of each pie diagram corresponds to sampling site coordinates (see Table 1); the area of each pie chart is proportional to total sample size (Table 1); the area of each slice in a pie chart is proportional to the number of samples for each species (Table 1). *V. kwatae* = black; *V. surinamensis* = gray; *V*. *michelii* = white

Leaf or cambium samples were collected from 93 *Virola* reproductive individuals belonging to several populations located in old, undisturbed stands of the eastern Guiana Shield (Table 1 and Figure 1). Sampled trees were separated by at least 100 m (to avoid sampling related trees) within each sampling site. We sampled small numbers of trees from a large number of sites for each species to cover as much as possible of the regional diversity for each species (Barthe et al., 2017). Whenever possible, we sampled stands where at least two of the three species co‐occur. In these areas, *terra firme* and bottomland habitats are intermingled, with habitat turnover typically occurring over shorter distances than seed dispersal and gene flow (Audigeos, Brousseau, Traissac, Scotti‐Saintagne, & Scotti, 2013; Brousseau, Bonal, Cigna, & Scotti, 2013). Botanical identification was performed on a subset of the samples (see Table 1) using the presence or absence of several specific characters (Sabatier, 1997); the remaining samples, although tagged with a putative species identity at sampling, were left unidentified for the time being. No recombined character assemblage or intermediate morphology was observed in the individuals with full botanical identification. For all samples, total genomic DNA was extracted according to the cetyltrimethylammoniumbromide (CTAB) procedure from Doyle and Doyle (1987) or an Invisorb DNA Plant HTS 96 kit (Stratec) according to the manufacturer's instructions. DNA quality was checked by spectrophotometry and by agarose gel electrophoresis.

**TABLE 1:** Sampling sites. The number of samples per species and per site is indicated. Numbers in parentheses indicate the number of samples with certified botanical identification (the remaining samples had putative identification in the field)

Sampling sites	Geographic coordinates	*V. michelii*	*V. surinamensis*	*V. kwatae*
Bafog	5°32′60.00″N–53°52′48.00″W	5 (5)	8 (8)	0
Paracou	5°16′45.70″N–52°55′23.50″W	3 (3)	2 (2)	0
Maroni	3°22′60.50″N–54°2′57.30″W	1	3	0
Mana	4°58′17.90″N–53°47′9.30″W	0	3	0
Oyapoque	3°42′26.40″N– 51°58′58.60″W	0	4 (1)	5
Trinité	4°40′10.60″N–53°16′60.00″W	1	5	0
Monts Emerilllons	3°15′36.00″N–53°11′24.00″W	3 (3)	0	3 (3)
Montagne Tortue	4°11′41.00″N–52°26′1.00″W	5	0	3
Saül	3°36′51.30″N–53°13′22.70″W	2	5	2
Piste St Elie	5°16′48.00″N–53°4′48.00″W	5 (5)	0	0
Saut Lavilette	4°6′55.80″N–52°12′31.40″W	5 (4)	0	0
MontagneTrésor	4°32′40.00″N–52°9′12.00″W	0	0	3 (1)
Approuague	4°2′28.70″N–52°40′29.70″W	2	2	13
Total		32	32	29

### 2.3 DNA amplification, sequencing, and genotyping

#### 2.3.1 Chloroplast DNA

We used two universal chloroplast sequence markers: *trnH‐psbA* and *trnC‐ycf6* (Shaw et al., 2005). PCRs were performed in a 15µl reaction volume containing 10 ng of DNA, 1× ThermoPolBuffer^®^ (New England Biolabs; 100 Mm KCl, 10 mM Tris‐HCl at Ph 7.4, 0.1 mM EDTA, 1 Mm dithiothreitol), 2.5 mM MgCl_2_, 0.2 mM each dNTP, 1 µM of each primer and 0.5 U New England Biolabs (New England Biolabs) ThermoPol^®^ Taq DNA polymerase, using the following thermal profile: 94°C for 4 min, 35 cycles of [45 s at 94°C, 45 s at 56°C, 1.5 min s at 72°C] and a final extension at 72°C for 8 min. PCR products were purified with ExoSAP‐IT (USB, Cleveland, OH). Sequencing reactions were performed in a 10 µl reaction volume containing either one or the other of the primers used in PCRs (2.5 µM), 5 × ABI Buffer^®^, 4 µl of 20‐fold diluted PCR product and BigDye Terminator^®^ v3.1 cycle sequencing kit (Applied Biosystems). Sequencing reactions were then purified on Sephadex columns (Millipore) following the manufacturer's instructions. Electrophoresis and detection were performed on an ABI 3130xl capillary sequencer (Applied Biosystems). DNA sequences were aligned with codoncode aligner v. 3.0 (CodonCode) and bioedit v. 5.0 (Hall, 1999). Both forward and reverse sequences were checked carefully by eye for all base‐calling errors. Alignments were adjusted manually to minimize the number of gaps. After base calling was completed, the sequences obtained on both strands were assembled using an R script (R Core Development Team, 2018) available from Dryad. Indels were removed from further analysis. Because in general no recombination occurs in chloroplast DNA, sequences of the two loci (*trnH‐psbA* and *trnC‐ycf6*) were assembled and treated as a single locus. Full sequences for both regions were obtained for 78 out of the 93 individuals originally sampled.

#### 2.3.2 Nuclear microsatellites

We applied nine nuclear microsatellites (nSSRs): *Vsur*34, *Vmul*2‐65, *Vseb*3, *Vsur*58, *Vseb*21, *Vsur*2‐35, *Vsur*2‐41, *Vmul*2‐66, and *Vmul*68 drawn from Draheim, Cui, and Dick (2009). PCRs were performed using 20 ng DNA in a 15 ml volume containing 1× ThermoPolBuffer^®^ (New England Biolabs; 100 Mm KCl, 10mM Tris‐HCl at pH 7.4, 0.1 mM EDTA, 1 Mm dithiothreitol), 2.5 mM MgCl_2_, 0.2 mM each dNTP, 0.2 µM of each primer and 1U New England Biolabs (New England Biolabs) ThermoPol Taq DNA polymerase. PCRs were carried out on an Applied Biosystems GeneAmp PCR System 9700 (Applied Biosystems) or a Bio‐Rad Peltier Tetrad DNA Engine thermal cycler (Bio‐Rad), using the following protocol: 94°C for 5 min, followed by 35 cycles of [30 s at 94°C, 40 s at 52°C, 1 min at 72°C] and a final extension at 72°C for 10 min. For each primer pair, the forward primer was labeled with fluorescent dyes (NED, PET, VIC, 6‐FAM). PCR products were separated and detected on an ABI 3130xl capillary sequencer (Applied Biosystems) with the Liz‐500 size standard. Genotypes were scored using the genemapper software package (Applied Biosystems).

### 2.4 Data analyses

#### 2.4.1 nSSR Bayesian cluster analysis and cpDNA haplotype network

To identify genetic clusters corresponding to botanical species, and to evaluate their genetic relationships, we analyzed the nine‐locus nSSR data set (full 93‐individual sample) using the Bayesian clustering method implemented in Structure version 2.0 (Pritchard, Stephens, & Donnelly, 2000) to infer the most likely number of genetic clusters *K* and to estimate cluster membership coefficients for each individual. Ten independent runs were performed for each *K* between 1 and 10, with an admixture model (to allow for individuals whose ancestry is in more than one species) and correlated allele frequencies, and without prior assumptions on sample identity, 200,000 MCMC iterations and a 50,000 iterations burn‐in period. We then selected the most plausible number of clusters *K* using both the approach based on ln*P*(*D*) (Pritchard et al., 2000) and the ∆*K*‐based approach (Evanno, Regnaut, & Goudet, 2005). Results from each batch of ten runs from the same *K* were summarized using CLUMPP (Jakobsson & Rosenberg, 2007), and the results were treated graphically with DISTRUCT (Rosenberg, 2004; two *V. michelii* and three *V. surinamensis* samples were discarded for clustering analyses because they had data for fewer than six nSSRs). We assigned individuals to a cluster if their admixture coefficient value was above the 0.90 threshold for a given inferred ancestral cluster. Following the “blind genetic survey” strategy (Duminil, Caron, Scotti, Cazal, & Petit, 2006), we used the specimens with firm botanical identification to assign Bayesian clusters to botanical species. All specimens having been left as “unidentified” or having a putative identification (see above), and assigned to a given cluster, were then taken as belonging to the botanical species corresponding to that cluster. To investigate relationships among chloroplast haplotypes, we constructed a phylogenetic network based on the method of statistical parsimony described by Templeton et al. using TCS version 1.16 (Templeton, Crandall, & Sing, 1992).

#### 2.4.2 Genetic diversity and genetic differentiation among species

For both the nine‐locus nuclear microsatellite data set and chloroplast DNA sequences, allelic richness (*A*), expected heterozygosity (*H*_e_), and pairwise *F*_ST_ values were computed on the clusters defined by Bayesian clustering using Arlequin 3.1 software (Excoffier, Estoup, & Cornuet, 2005); haplotype diversity (*H*) and haplotype richness (*N*_h_) were also computed for chloroplast data only. Significance of differentiation was tested by permutation (*N* = 1,000; Excoffier, Smouse, & Quattro, 1992). Since no genetic maps exist for *Virola*, we tested for the presence of linkage disequilibrium among our genetic markers by means of GENEPOP (Raymond & Rousset, 1995).

#### 2.4.3 Coalescent modeling

**FIGURE 2:**
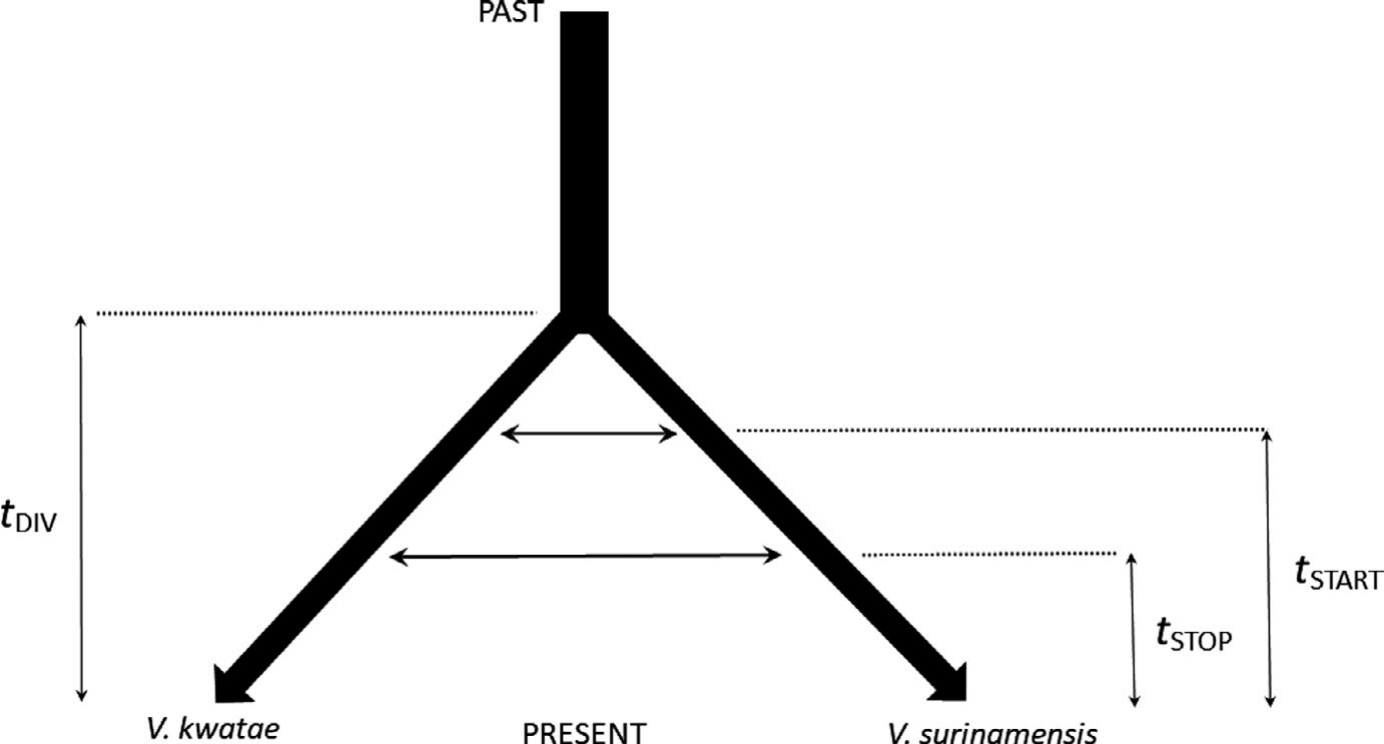
Diagram of the two‐species coalescent model analyzed here, with the identification of model parameters: *t*_DIV_, time (in generations) of species divergence; *t*_START_, time (in generations) of start of gene flow; *t*_STOP_, time (in generations) of stop of gene flow; horizontal double arrows indicate gene flow from *V. kwatae* to *V. surinamensis* and from *V. surinamensis* to *V. kwatae*

We used a combination of coalescent modeling and ABC parameter estimation to test whether gene flow occurs, or occurred, between *V*. *surinamensis* and *V*. *kwatae* clusters. *V*. *michelii* was not included in coalescent modeling because the strong level of genetic divergence with the two other species suggests a much older speciation event, which was not contemporary to the *V*. *surinamensis*/*V*. *kwatae* pair. The speciation process was modeled by the following historical events (from past to present, see Figure 2): the time of divergence between the two species, *t*_DIV;_ the starting time of a gene flow event, *t*_START_; the ending time of gene flow, *t*_STOP_. Genotype data at nine nuclear microsatellites were simulated using the fastsimcoal (Excoffier & Foll, 2011) program. The priors used to perform 1,500,000 coalescent simulations are as follows. We simulated nSSRs using a generalized stepwise mutation model (GSM; Estoup, Jarne, & Cornuet, 2002; Zhivotovsky, Feldman, & Grishechkin, 1997) with two parameters: the mean mutation rate (*µ*) and the mean of the geometric distribution of the probability of being a one‐step mutation (*P*) were drawn respectively from Uniform [10^–5^; 10^–3^] and Uniform [0.1; 0.3] hyperprior distributions. Demographic parameters were drawn with the following distributions: Log‐Normal [100; 10^7^] for current or ancestral effective population sizes; directional migration rate per generation: uniform [10^–4^; 0] for both directions; we drew event times (time of population divergence (*t*_DIV_), time of start of gene flow (*t*_START_), and time of stop of gene flow (*t*_STOP_)) in generations, from a Uniform [1; 10^5^] distribution; other priors were as in Barthe et al. (2017).

Approximate Bayesian Computation (ABC; Bertorelle et al., 2010; Csilléry et al., 2010) was used to estimate model parameters. Eighteen summary statistics were computed: for each population, mean and standard deviation of number of alleles, mean and standard deviation of heterozygosity, mean and standard deviation of Garza–Williamson index (the ratio of number of alleles to allelic range), mean and standard deviation of allelic range, together with pairwise *F*_ST_ and Δ*μ* ^2^ (Goldstein, Linares, Cavalli‐Sforza, & Feldman, 1995). These summary statistics were chosen for the calculations of posteriors parameters because they are sensitive to variation in migration rates (Bohonak, 1999). All summary statistics were computed using Arlequin 3.5 (Excoffier & Lischer, 2010) and its companion ARLSUMSTAT. Calculations of posterior distributions of parameters were performed using the “abc” R package (Csilléry, François, & Blum, 2012). Parameters were estimated on the 0.01 fraction of the best simulations, based on Euclidean distances from the observed data in summary statistics multidimensional space (Beaumont, Zhang, & Balding, 2002), by neural networks algorithms. The accuracy of the estimates was checked by both the “goodness‐of‐fit” test of the “abc” R package and PCA analysis. Parameter posterior estimates were interpreted as follows to reconstruct historical patterns of gene flow: if *t*_DIV_ = *t*_START_, we are in presence of divergence with gene flow; if *t*_DIV_ > *t*_START_, we are witnessing a secondary contact event; if *t*_START_ roughly equals *t*_STOP_, a gene flow event was not protracted in time; if *t*_START_ > *t*_STOP_, gene flow has been going on for a long period of time. In addition, we built two additional composite parameters: the difference D*T*_1_ between *t*_DIV_ and *t*_START_ and the difference D*T*_2_ between *t*_START_ and *t*_STOP._ It is important to note that all parameters are scaled to mutation rate (locus^−1^ generation^−1^), which is also estimated in the model. Absolute value should not be taken as strictly informative; ratios, differences, and comparisons are, on the contrary, entirely meaningful.

## 3 RESULTS

### 3.1 Genetic variability

**FIGURE 3:**
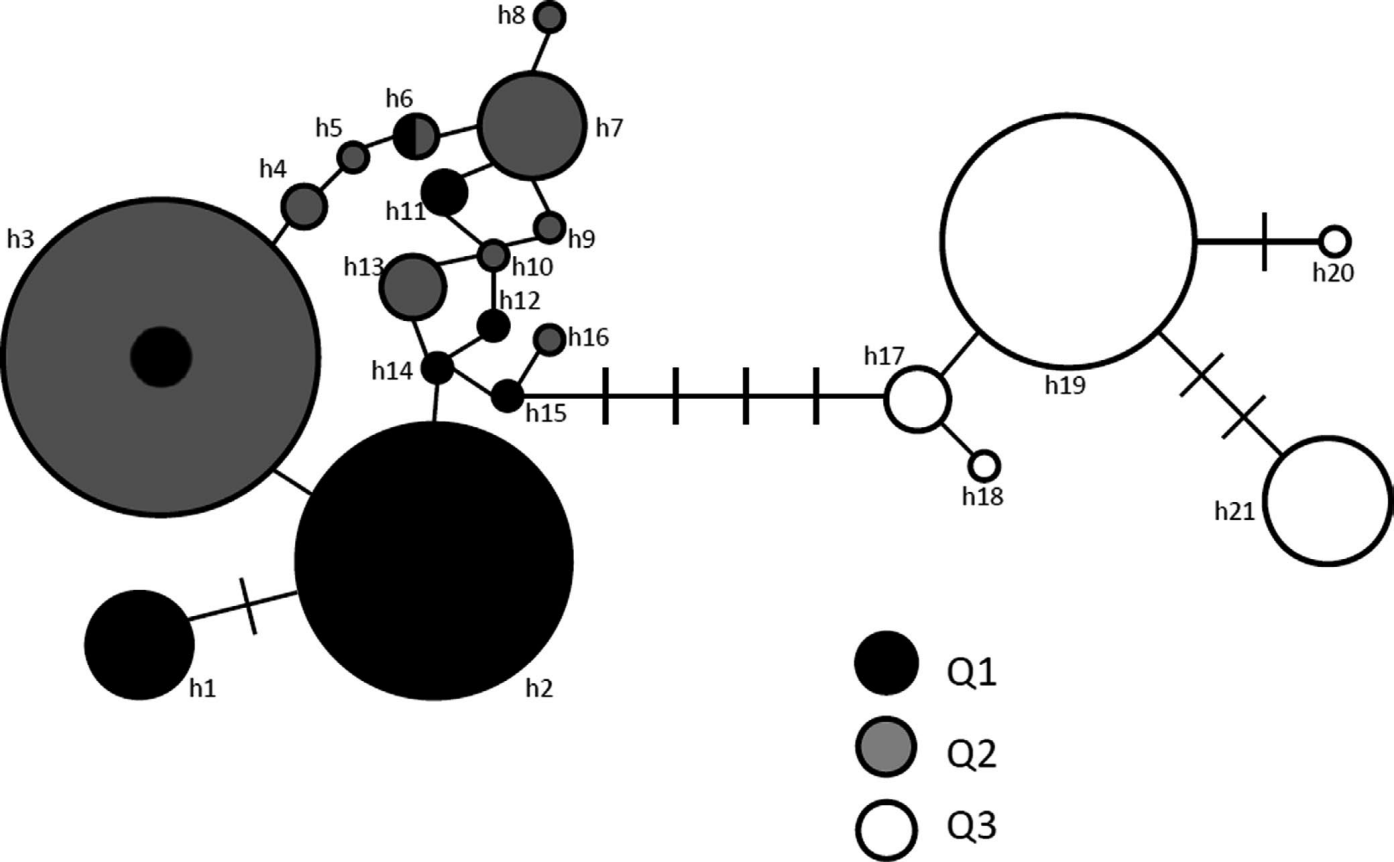
Chloroplast haplotype network. Disk sizes represent the number of copies of each haplotype in the sample. The number of segments between two disks represents the number of mutational steps that separate the corresponding haplotypes. Each color corresponds to a Bayesian cluster: black/*V. kwatae*; gray/*V. surinamensis*; white/*V. michelii*

All the ten nuclear microsatellites were polymorphic in all clusters/species. Allelic richness (averaged over loci) varied between 14.0 for *V. kwatae* and 17.7 for *V. surinamensis* (Table 2). Expected heterozygosity values ranged between 0.82 for *V. michelii* and *V. kwatae* to 0.89 for *V. surinamensis* (Table 2). Nineteen polymorphic sites were observed globally for *trnH‐psbA* and *trnC‐ycf6* sequences. These variants combined into twenty‐one haplotypes: eight for *V. kwatae* (haplotypes h1–h3, h6, h11, h12, h14, and h15), 10 for *V. surinamensis* (haplotypes h3–h10, h13, and h16), and five for *V. michelii* (haplotypes h17–h21). Two haplotypes (h3 and h6) were shared between the two most closely related clusters/species, and haplotypes belonging to these clusters/species were interspersed in the haplotype network; in contrast, haplotypes of *V. michelii* were distinct from those of the remaining clusters, from which they were separated by at least five mutational steps (Figure 3). Haplotype richness was as follows: *A* = 10, 8 and 5, and chloroplast diversity values were *H* = 0.21, 0.25, and 0.25 (Table 2) for *V. kwatae, V. surinamensis,* and *V. michelii,* respectively.

**TABLE 2:** Diversity parameters assessed with nuclear microsatellites and chloroplast DNA markers in *Virola* Bayesian clusters (kwa/ *Virola kwatae*; sur/ *Virola surinamensis*; mic/ *Virola michelii*)

Species	Nuclear microsatellites (7 loci)			Chloroplast DNA		
	*2N*	*A*	*H*_e_	*N*	*N*_h_	*H*
*kwa*	52	14.0 (7.5)	0.82 (0.19)	26	8	0.21 (0.09)
*sur*	60	17.7 (6.8)	0.89 (0.08)	30	10	0.25 (0.17)
*mic*	50	16.6 (6.4)	0.82 (0.25)	25	5	0.25 (0.16)

Note

*N*, number of samples; *A*, allelic richness; *H*_e_, Nei's expected genetic diversity; *N*_h_, haplotype richness; *H*, haplotypic genetic diversity (i.e., Nei's genetic diversity computed on haplotype frequencies). Standard errors are shown in parentheses.

No evidence for linkage disequilibrium among the microsatellite markers used in this study was found (data not shown): out of 36 pairwise comparisons, only one gave a significant result, which we interpret as Type I error.

**FIGURE 4:**
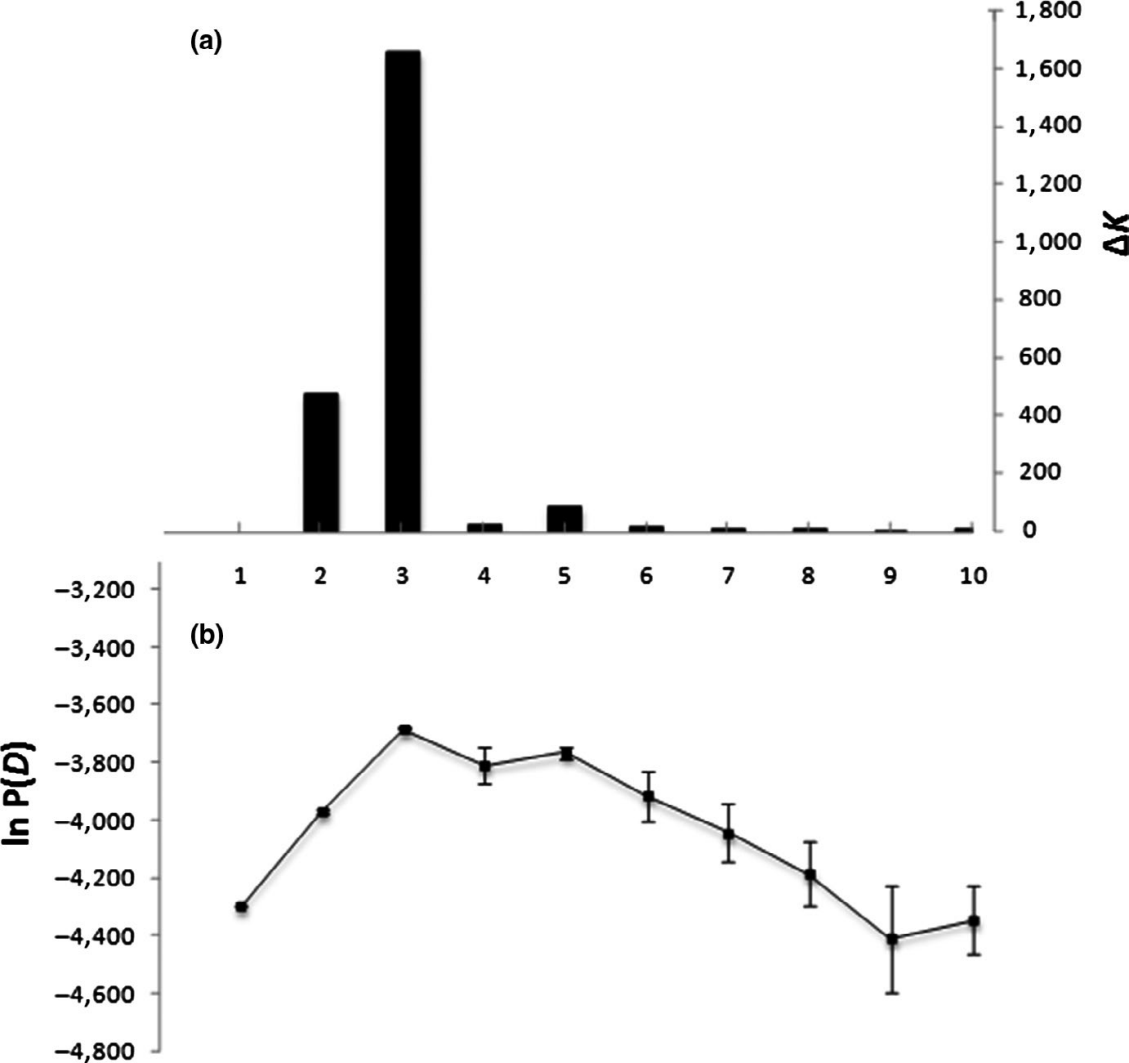
Identification of the best number of groups (*K*) in the automated Bayesian assigned as performed by structure. (a) Values of Evanno's Δ*K* statistic (*y*‐axis) as a function of *K* (*x*‐axis); (b) values of the logarithm of posterior probabilities (ln *P*(*D*), *y*‐axis) as a function of *K* (*x*‐axis). In both cases, the best value is *K* = 3

**FIGURE 5:**
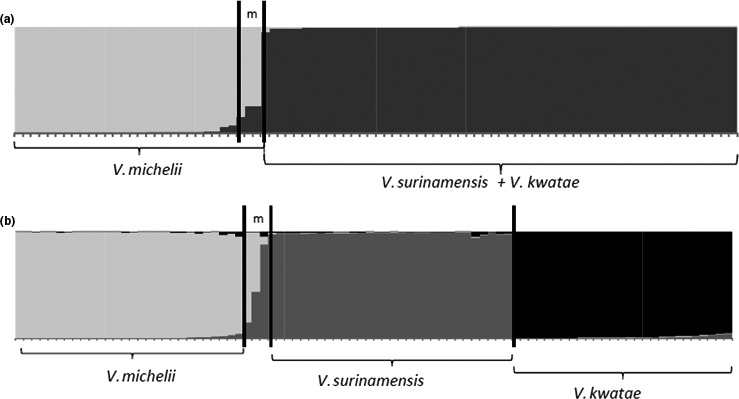
Individual *Q* values (i.e., probabilities of assignment to a given group) obtained in the automated Bayesian assigned as performed by structure. Although the best *K* value is, unequivocally, *K* = 3, we also report here *Q* values for *K* = 2, to highlight the proximity of the clusters corresponding to *V. surinamensis* and *V. kwatae*. (a) *Q* values for *K* = 2; (b) *Q* values for *K* = 3

In the automated Bayesian assignment analysis performed by STRUCTURE, the highest posterior probability was obtained for *K* = 3 genetic clusters according to the ad hoc *∆K* statistics (Evanno et al., 2005; Figure 4a; *∆K* = 1,664.65). This result is also confirmed by the values of ln*P*(*D*) (Figure 4b, with ln*P*(*D*) = −3681,78 for *K* = 3). At *K* = 3 (Figure 5b), *V. kwatae*, *V. surinamensis*, and *V. michelii* grouped into three well‐separated clusters. The Bayesian clusters did not show any mixing of botanically certified specimens: that is, each Bayesian cluster contained certified samples of only one species. Therefore, we could confidently assign Bayesian clusters to botanical species. Three samples showed signs of introgression (Figure 5), of which two (M671 and M257) were botanically certified specimens (*V. michelii*) and the third (BD64) had a typical *V. surinamensis* chlorotype. The former ones were assigned to the cluster containing all other certified *V. michelii* specimens, and the latter to the species to which its chlorotype was associated (*V. surinamensis*).

Differentiation was significant for all pairs of clusters/species (Table 3) for both nuclear and chloroplast markers, and was larger, for both kinds of markers, between *V. kwatae* and *V. michelii* (0.165 and 0.782 for nuclear and chloroplast markers, respectively) and between *V. surinamensis* and *V. michelii* (*F*_ST_ = 0.131 and 0.785) than between *V. surinamensis* and *V. kwatae* (0.080 and 0.268). In the chloroplast haplotype network (Figure 3), *V. surinamensis* and *V. kwatae* haplotypes are intermingled (with haplotypes h3 and h6 occurring in both species). Taken together with the results of the Bayesian analysis and differentiation values, this suggests that formerly assumed phylogenetic proximity of *V. michelii* and *V. kwatae* is unsupported and that *V. surinamensis* and *V. kwatae* are sister species.

**TABLE 3:** Pairwise *F*_ST_ values of genetic differentiation among the three genetic clusters for chloroplast DNA markers (below) and nuclear microsatellites (above) as obtained *without introgressed individuals*. (*kwa*/*Virola kwatae*; *sur*/*Virola surinamensis*; *mic*/*Virola michelii*)

	*kwa*	*sur*	*mic*
*kwa*	–	0.082	0.166
*sur*	0.262	–	0.133
*mic*	0.782	0.783	–

Note

All estimates were significantly different from zero at the *α* = 5% significance level (*p*‐value < .05 for all pairs).

**FIGURE 6:**
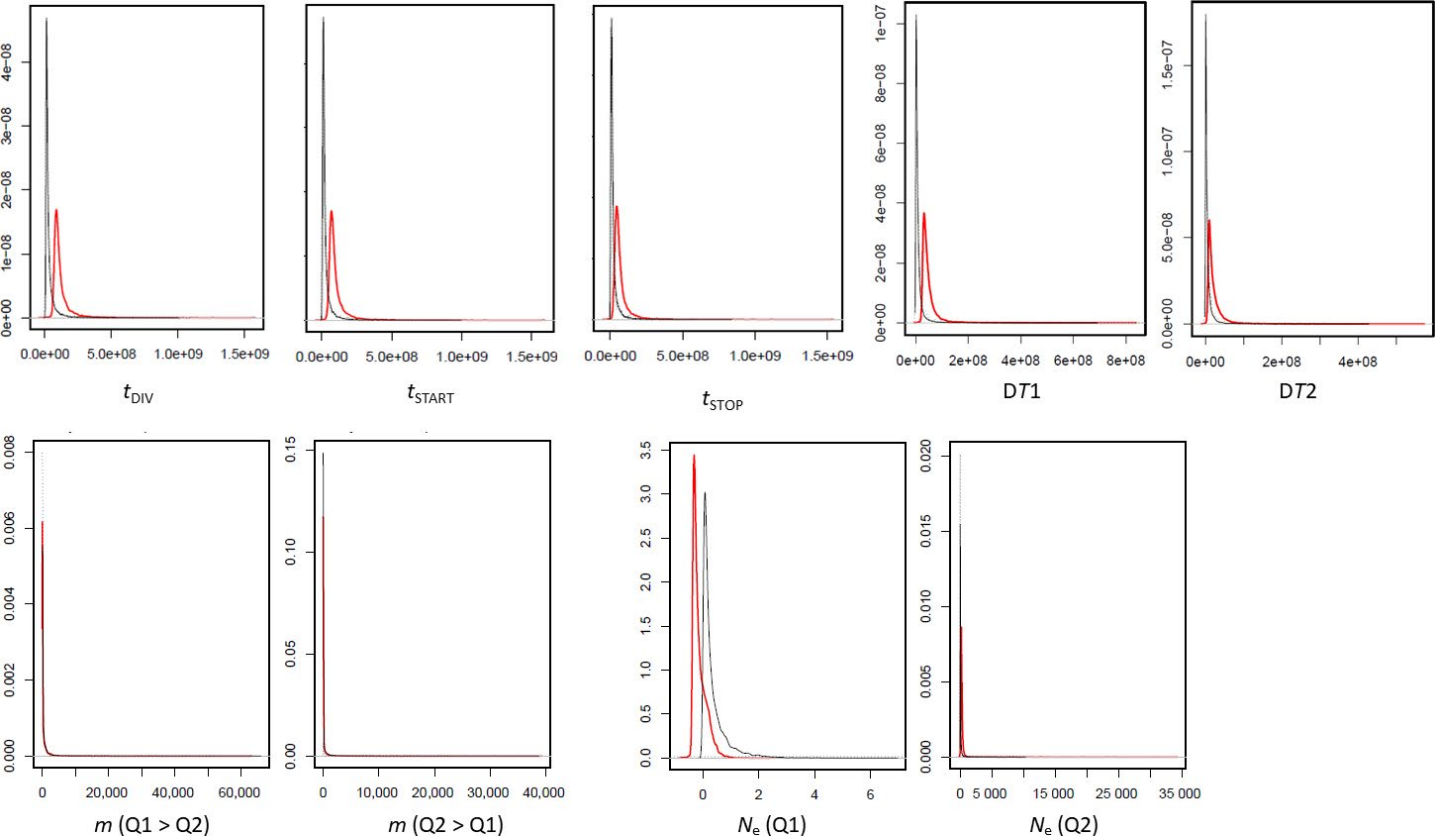
Prior and posterior probability density for coalescent parameters. Thick, red lines: posteriors; thin, gray lines: priors. All plots, *y*‐axis: probability density. On the *x‐*axis (all parameters scaled by *μ*): *t*_DIV_, time of divergence between *V. surinamensis* and *V. kwatae*; *t*_START_, starting time of gene flow; *t*_STOP_, ending time of gene flow. D*T*_1_ (composite parameter), difference between *t*_DIV_ and *t*_START_; D*T*_2_ (composite parameter), difference between *t*_START_ and *t*_STOP_; *m (Q*_1_ > *Q*_2_), gene flow from *V. kwatae* to *V. surinamensis*; *m (Q*_1_ > *Q*_2_), gene flow from *V. surinamensis* to *V. kwatae*; *N*_e_ (*Q*1), effective population size for *V. kwatae*; *N*_e_ (*Q*2), effective population size for *V. surinamensis*

**FIGURE 7:**
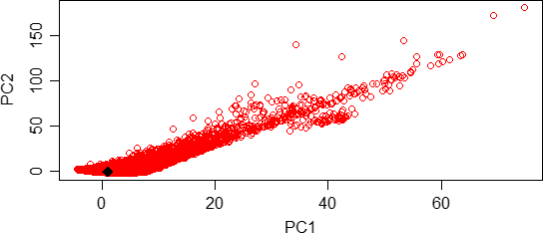
PCA analysis. The vector of empirical summary statistics (blue diamond) falls within the space of the simulated summary statistics

Coalescent/ABC analyses were applied to the *V. kwatae*/*V. surinamensis* pair. Table 4 reports the medians of the posterior distributions for population parameters estimated under the ABC framework. Posterior distributions of the simple and composite (scaled) event time parameters, that is, *t*_DIV_, *t*_START,_ and *t*_STOP_, plus the differences in time between *t*_START_ and *t*_DIV_ (D*T*_1_) and between *t*_STOP_ and *t*_START_ (D*T*_2_) scaled by the mutation rate, display well‐defined peaks, distinct from the priors (Figure 6), thus indicating that our data set was informative relative to the estimation of the parameters. In the goodness‐of‐fit test, the probability of a nonrandom deviation of the estimated parameters from the observed was *p* = .21. In the principal component analysis, the vector of empirical summary statistics fell well within the cloud of s simulated summary statistics (Figure 7).

**TABLE 4:** Summary of posterior distributions for population parameters estimated under the ABC frameworks

	*t*_DIV_	*t*_START_	*t*_STOP_	D*T*_1_	D*T*_2_	*m (kwa > sur)*	*m (sur > kwa)*	*N*_e_ (kwa)	*N*_e_ (*sur*)
95% CI lower limit	6.79e + 07	5.11e + 07	3.01e + 07	2.14e + 07	1.57e + 07	3.20e−03	−5.00e−04	−7.25e−02	1.07e + 02
Median	1.01e + 08	8.55e + 07	5.78e + 07	3.60e + 07	2.43e + 07	9.80e−03	6.00e−04	5.38e−02	2.74e + 02
95% CI upper limit	3.67e + 08	3.32e + 08	2.54e + 08	1.45e + 08	9.89e + 07	7.78e−01	4.14e−01	9.19e−01	8.07e + 02

Note

All values must be considered as scaled to mutation rate. *kwa*/*Virola kwatae*; *sur*/*Virola surinamensis*. Note that all parameters are actually scaled to mutation rates, which are themselves estimated from the model; only comparisons should be considered relevant, but not absolute values.

Abbreviations: D*T*_1_, difference between *t*_START_ and *t*_DIV_; D*T*_2_, difference between *t*_STOP_ and *t*_START_; *m* (*kwa* > *sur*), gene flow (genes genes^−1^ generation^−1^) from *kwa* to *sur*; *m *(*sur* > *kwa*), gene flow (genes genes^−1^ generation^−1^) from *sur* to *kwa*; *N*_e_ (*kwa*), effective population size for *kwa*; *N*_e_ (*sur*), effective population size for *sur*; *t*_DIV_, time (generations) to the divergence of *kwa* and *sur*; *t*_START_, time (generations) to the beginning of gene flow between *kwa* and *sur*; *t*_STOP_, time (generations) to the end of gene flow between *kwa* and *sur*.

Scaled effective population size was much higher for *V. surinamensis* than for *V. kwatae*, with medians 2.74 × 10^2^ and 5.38 × 10^−2^, respectively, with a four orders‐of‐magnitude difference (Table 4). Estimated times of species divergence, start of gene flow, and stop of gene flow are different from each other as shown (Table 4) by the (scaled) posterior credible intervals and medians of the corresponding simple parameters. Regarding composite parameters, D*T*_1_ (the time elapsed between speciation and the onset of gene flow) is positive (with median an order of magnitude smaller than *t*_DIV_), indicating that there was no gene flow at speciation, but that it occurred later probably during a secondary contact; gene flow went on for a certain amount of time, as indicated by D*T*_2_, the time elapsed between the onset and the arrest of gene flow, and then stopped sometime in the past (*t*_STOP_). If we take median estimators as a reference (Table 4), the two species would have spent, very roughly, a quarter of their postdivergence biological history without gene flow, then another quarter with, and then again a half without. Notice that, by construction (Figure 2), *t*_STOP_ + D*T*_2_ + D*T*_1_ = *t*_DIV_. As a proof of the method of estimation, if we sum median estimators of the terms on the left hand of the equality, we obtain 5.78 × 10^7^ + 3.60 × 10^7^ + 2.43 × 10^7^ = 1.18 × 10^8^, not very different from the median estimator of *t*_DIV_ (1.01 × 10^8^), which is the right‐hand side of the equality. Gene flow was asymmetric, with larger flow from *V. kwatae* to *V. surinamensis* than the other way round.

## 4 DISCUSSION

A combination of analyses on nuclear SSR markers and chloroplast sequences allowed us to infer the genetic relationships among three sympatric species in the *Virola* genus and to propose a model for the history and strength of interspecific gene flow.

Based on our data, phylogenetic reconstruction indicates that the most recently described species, *V. kwatae* (previously undistinguished from *V. michelii*), has possibly diverged from *V. surinamensis*, in spite of its ecological and morphological similarity to *V. michelii*.

We then focused on the evolutionary relationships between the two putative sister species. The smaller (scaled) effective population size in *V. kwatae* than *V. surinamensis*, estimated by coalescent analysis, is in agreement with the relative size of the species' ranges. The main outcome of coalescent modeling is that the two species underwent intermittent genetic exchange throughout their postdivergence history, with gene flow starting and then stopping at least once in the geological past. This appears to be a rather common phenomenon in species‐rich Amazonian tree genera: Caron et al. (2019) show that many congeneric species share genes by introgression; the results presented here suggest that this is not necessarily the result of ongoing gene flow. Nevertheless, the few individuals, which are apparently introgressed between *V. surinamensis* and *V. michelii* (Figure 5b), are an indication that interspecific mating can currently occur. Hybridization between *Virola* species is theoretically possible, because of their overlapping flowering times (Loubry & Puig, 1994; Sabatier, 1997) and because turnover between the habitats they occupy occurs over short distances (few tens to few hundred meters) relative to dispersal distances, which can be large for pollen and seeds in tropical trees (Dick, Etchelecu, & Austerlitz, 2003) and particularly in some *Virola* species (Hardy et al., 2006). In addition, flower morphologies are similar, and at least, *V*. *surinamensis* is pollinated by multiple generalist pollinators (Gonçalves Jardim & Gomes da Mota 2007). Individual BD64 could be interpreted as an early‐generation hybrid (Pritchard et al., 2000), suggesting that interspecific mating is possible, even though our analyses were not specifically designed to detect hybridization; however, current mating events do not seem to contribute to gene flow, which—according to our coalescent model—has stopped far back in the past. The clear ecological divergence between the two species and their overlapping distribution in the Eastern Guiana shield would a priori suggest a process of ecological speciation (Schluter, 2001), in which endemic *V. kwatae* would have sympatrically diverged from widespread *V. surinamensis*; yet on the contrary, the posterior distributions of event times and of composite parameters D*T*_1_, D*T*_2_ support intermittent secondary contact: D*T*_1_ >> 0 suggests that gene flow did not begin until long after divergence, and D*T*_2_ >> 0 suggests that gene flow stopped long time ago. Therefore, our modeling results support a scenario of allopatric speciation, followed by fluctuating secondary contact: There may actually have been more than one stop‐and‐go events, but our data probably lack the necessary resolution to detect them. Speciation with continuous gene flow is possible (Dettman, Sirjusingh, Kohn, & Anderson, 2007; Kondrashov & Kondrashov, 1999; Rice & Hostert, 1993) and it has been theoretically (see Nosil & Crespi, 2004) and in a natural scenario (Lin, Ziegler, Quinn, & Hauser, 2008) shown between populations of the same species and at the interspecific level (Kremer et al., 2002; Whittemore & Schaal, 1991), but unambiguous examples of sympatric speciation are rare in nature (Coyne & Orr, 2004)_;_ many cases of divergence with gene flow discussed in the literature involve a phase of allopatry, especially in geographic zones where populations have been isolated for a long time, for example, during glaciations. The case we report here appears to belong to the latter type.

Similar levels of interspecific genetic differentiation have been observed in ecologically diverse species complexes, and particularly in oaks, with *F*_ST_ varying between 0.011 and 0.378 between *Q. petraea* and *Q. robur*, which also occupy different (dry vs. wet) habitats (Neophytou, Aravanopoulos, Fink, & Dounavi, 2010), and have possible undergone cycles of geographical connection and separation through glacial cycles (Leroy et al., 2017). Could it be that the patterns of intermittent gene flow we observe in the *Virola* genus have been determined by climatic events? To tentatively answer this question, we can attempt a crude estimation of the timing of events reported here. If we assume an average value for SSR mutation rates of 10^–4^ and a generation time of 50 years (Barthe et al., 2017), we estimate the following times in years: *t*_DIV_ = 505,000; *t*_START_ = 428,000; *t*_STOP_ = 289,000 (example: *t*_STOP_ = *t*_STOP_/*µ* = 5.78 × 10^7^/10^−4^ = 5,780; this, multiplied by 50, yields 289,000 years). These estimates of divergence time and of onset and stop of gene flow between *V. surinamensis* and *V. kwatae* support the hypothesis that these events occurred during the Pleistocene climatic cycles, perhaps due to cycles of disjunction and merger of their ranges and/or their habitats (Bush & de Oliveira, 2006; Colinvaux, De Oliveira, & Bush, 2000; Rull, 2013). In summary, our study highlights an example of interspecific gene flow between ecologically divergent tree species, well‐fitting with the suggestion by Ellstrand (2014) that plant species form meta‐populations that exchange genes with variable, asymmetric rates. Given the relatively small number of neutral genetic markers used, we cannot make inferences about several important points, such as whether the amount of gene flow differs among genomic regions, or whether it involves regions of relevant adaptive value, which would be of great interest in the dissection of the relative weight of selection and gene flow on divergence patterns across the genome (in the *Picea mariana*/*Picea rubens* pair, for example, not introgressed or “impermeable” genomic regions coexist with highly introgressed, or “permeable”, ones (de Lafontaine & Bousquet, 2017)). Introgression is emerging as an important source of adaptation as proven by the large number of studies recently published (Suarez‐Gonzalez, Lexer, & Cronk Quentin, 2018, for a review).

Significant (past) gene flow detected between *V. surinamensis* and *V. kwatae* is consistent with phylogenetic radiation without strict reproductive isolation, a property of both ecological speciation and mechanisms of maintenance of genetic integrity in species complexes. Both models may represent suitable mechanisms to explain the great functional, genetic and specific diversity observed in stable, continuous biomes such as Amazonian forests.

## CONFLICT OF INTEREST

None declared.

## AUTHOR CONTRIBUTION

**Giorgio Binelli:** Formal analysis (equal); Investigation (equal); Writing‐original draft (equal); Writing‐review & editing (equal). **William Montaigne:** Data curation (equal); Investigation (equal). **Daniel Sabatier:** Conceptualization (equal); Data curation (equal); Investigation (equal); Supervision (equal). **Caroline Scotti‐Saintagne:** Conceptualization (equal); Formal analysis (equal); Investigation (equal); Writing‐original draft (equal); Writing‐review & editing (equal). **Ivan Scotti:** Conceptualization (equal); Formal analysis (equal); Funding acquisition (equal); Methodology (equal); Writing‐original draft (equal); Writing‐review & editing (equal).

## Data Availability

Original nuclear SSR and chloroplast sequence data, along with provenance, species identification and identification status of each voucher, have been deposited on DRYAD (https://doi.org/10.5061/dryad.vp65h).
